# Targeting the TAM Receptors in Leukemia

**DOI:** 10.3390/cancers8110101

**Published:** 2016-11-08

**Authors:** Madeline G. Huey, Katherine A. Minson, H. Shelton Earp, Deborah DeRyckere, Douglas K. Graham

**Affiliations:** 1 Aflac Cancer Center of Children’s Healthcare of Atlanta, Department of Pediatrics, Emory University, Atlanta, GA 30322, USA; madeline.huey@emory.edu (M.G.H.); katherine.minson@emory.edu (K.A.M.); deborah.deryckere@emory.edu (D.D.); 2 UNC Lineberger Comprehensive Cancer Center, Departments of Medicine and Pharmacology, University of North Carolina, Chapel Hill, NC 27514, USA; shelton_earp@med.unc.edu

**Keywords:** TYRO3, AXL, MERTK, Gas6, leukemia, multiple myeloma, tyrosine kinase inhibitor, resistance, hematopoiesis, signaling pathways

## Abstract

Targeted inhibition of members of the TAM (TYRO-3, AXL, MERTK) family of receptor tyrosine kinases has recently been investigated as a novel strategy for treatment of hematologic malignancies. The physiologic functions of the TAM receptors in innate immune control, natural killer (NK) cell differentiation, efferocytosis, clearance of apoptotic debris, and hemostasis have previously been described and more recent data implicate TAM kinases as important regulators of erythropoiesis and megakaryopoiesis. The TAM receptors are aberrantly or ectopically expressed in many hematologic malignancies including acute myeloid leukemia, B- and T-cell acute lymphoblastic leukemia, chronic lymphocytic leukemia, and multiple myeloma. TAM receptors contribute to leukemic phenotypes through activation of pro-survival signaling pathways and interplay with other oncogenic proteins such as FLT3, LYN, and FGFR3. The TAM receptors also contribute to resistance to both cytotoxic chemotherapeutics and targeted agents, making them attractive therapeutic targets. A number of translational strategies for TAM inhibition are in development, including small molecule inhibitors, ligand traps, and monoclonal antibodies. Emerging areas of research include modulation of TAM receptors to enhance anti-tumor immunity, potential roles for TYRO-3 in leukemogenesis, and the function of the bone marrow microenvironment in mediating resistance to TAM inhibition.

## 1. Introduction

Leukemia and other lymphoid neoplasms, make up the fifth most common cancer type in the United States, with an estimated 162,020 new cases and approximately 56,630 deaths due to the disease per year [1]. Current intensive cytotoxic therapies are highly toxic and associated with short and long term side effects that are often prohibitive in older patients. The application of personalized medicine and targeted therapies such as tyrosine kinase inhibitors (TKI) has prolonged survival and reduced therapy-associated toxicity for some leukemia patients, but development of resistance is a significant problem. In addition, few targeted options for treatment of AML (acute myeloid leukemia) are available and prognosis for these patients is particularly poor with a five year overall survival of only 26.6% compared to 59.7% for patients with all types of leukemia [2]. Thus, new targets and therapies for leukemia treatment are needed to provide safer, more effective options for patients with leukemia and this need is particularly urgent for patients with AML. Ideally these therapies will be efficacious alone and extremely potent in combination with other therapeutics. These properties could allow for increased therapeutic efficacy and potentially dose de-escalation of cytotoxic chemotherapies while still achieving durable remissions, such that patients who cannot tolerate high doses of chemotherapy could be successfully treated with a greatly reduced risk for long-term side effects. In addition, treatment with multiple targeted agents in combination could decrease the incidence of resistance relative to single agent therapies.

TYRO3, AXL, and MERTK comprise the TAM family of receptor tyrosine kinases (RTKs). Several TAM RTK ligands have been described, including Gas6 and Protein S, and they differentially activate the TAM family members [3,4]. Gas6 binds to all three TAM receptors with the highest affinity for AXL [5,6]. Similarly, Protein S mediates more potent activation of TYRO3 compared to Gas6, MERTK is activated by either ligand, and AXL is not responsive to Protein S. Activation of the TAM receptors is greatly increased in the presence of phosphatidylserine (PS) and calcium, as the optimal TAM RTK ligand in most cases is a complex of the protein ligand (e.g., Gas6, Protein S) and PS on apoptotic cells [6,7].

MERTK and AXL have been implicated in numerous hematopoietic malignancies, including acute leukemias (AML and acute lymphoblastic leukemia—ALL), chronic leukemias (chronic myeloid leukemia—CML and chronic lymphocytic leukemia—CLL), and multiple myeloma (MM). While TAM kinases are not strong drivers of proliferation like more traditional oncogenes, their aberrant or ectopic expression in leukemia cells activates pro-survival signaling to promote tumor cell survival. Thus, TAM kinase functions are most important under conditions of cell stress and may be particularly critical for tumor cell survival in the context of leukemia therapy. Multiple mutations in TAM kinases have been reported, but have not been fully characterized [6,8]. However, at least one reported translocation of MERTK, leading to production of a TMEM87B-MERTK fusion protein, transforms Ba/F3 cells to an IL3-independent phenotype. As prognostic factors, AXL and its ligand Gas6 have been identified as predictors of poor outcome in AML [9,10]. These and other data identify TAM RTKs as potential therapeutic targets in numerous hematopoietic malignancies.

In this review, we will discuss the TAM family of kinases in the context of leukemia, the mechanisms by which TAM RTKs promote cancer cell survival and tumorigenesis, and roles for TAM kinases in resistance to targeted agents and cytotoxic chemotherapies. Finally, as TYRO3, AXL, and MERTK are attractive therapeutic targets in leukemia, we will discuss recent advances toward introduction of small molecule inhibitors of MERTK and AXL into the clinic.

## 2. TAM Receptors in Normal Hematopoeisis

TAM RTKs have a vast array of physiologic functions; the most well described being their roles in innate immune control, NK cell differentiation, efferocytosis, clearance of apoptotic debris, and hemostasis. These aspects of TAM RTK function have been extensively reviewed elsewhere [6,11,12,13,14]. There is increasing evidence that TAM RTKs and their ligand, Gas6, are also critical in maintaining normal hematopoiesis, particularly in the erythroid and megakaryocytic lineages. Here we review these aspects of TAM RTK expression and function.

### 2.1. TAM RTK Expression in the Hematopoietic System

**Figure 1:**
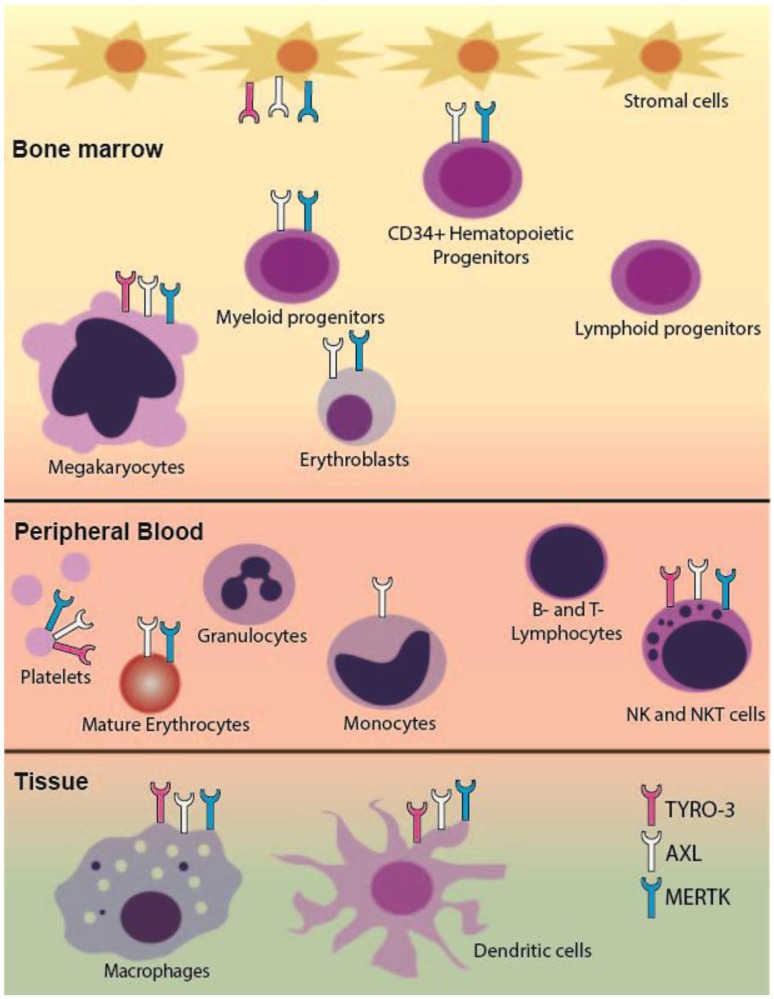
Schematic of TAM receptor expression in hematopoietic cells. Differential expression of TAM receptors in the hematopoietic system is shown. AXL protein and mRNA are expressed in bone marrow stromal cells. MERTK and AXL are expressed in CD34^+^ hematopoietic progenitors and some stages of myeloid development including erythrocyte precursors in the bone marrow. All TAM receptors are present in megakaryocytes, mature platelets, tissue macrophages, and dendritic cells. MERTK is expressed in bone marrow monocytic precursors and tissue macrophages but not in circulating monocytes. Although TAM receptors are not expressed in lymphoid progenitors or mature lymphocytes, they are highly expressed in NK and NKT cells.

Within the hematopoietic system, TAM RTKs are expressed primarily on myeloid lineage cells, with the notable exception of expression on NK and NKT cells in the lymphoid lineage (Figure 1) [6,11,15,16,17]. All three receptors are normally expressed on macrophages, megakaryocytes, and dendritic cells. While MERTK is present in CD11b^+^ bone marrow cells and is highly expressed in tissue macrophages, there is minimal MERTK in circulating normal monocytes [6,15,18]. Interestingly, MERTK expression in circulating monocytes is upregulated in certain inflammatory states such as acute and chronic liver failure [19], lupus [20], and septic shock [21]. Expression of both AXL and MERTK are increased in monocytes upon induction of differentiation to macrophages [16,17]. Within the erythroid lineage both AXL and MERTK are expressed, however TYRO3 expression is less well defined [22,23]. Notably, expression of AXL and MERTK is absent in mature granulocytes and in all stages of lymphocyte development [15,16,17,24]. AXL protein expression has been demonstrated in bone marrow stromal cells [16,25] and interestingly, by transcript level TAM RTKs are among the cell signaling receptors most differentially expressed in bone marrow mesenchymal stromal cells when compared to bone marrow cell-derived hematopoietic stem/progenitor cells [26].

### 2.2. Regulation of Erythropoiesis

TAM receptors and their ligand, Gas6, play a critical role in erythropoiesis in mice [22,23]. Normal erythropoiesis is characterized by progression through five distinct stages of erythroid development—erythroid progenitors, proerythroblasts, basophilic erythroblasts, polychromatophilic erythroblasts, and orthochromatophilic erythroblasts. Both AXL and MERTK are expressed throughout erythroid development (Figure 1) [22] and TYRO3 mRNA and protein are expressed in murine splenic erythroblasts [23]. Interestingly, AXL^−/−^MERTK^−/−^ double knockout mice accumulate erythroid progenitors in the spleen, likely due to a block in proerythroblast development. Additionally, in models of acute hemolysis, while MERTK^−/−^ single knockout mice are able to fully recover to normal hematocrit levels, AXL^−/−^MERTK^−/−^ and AXL^−/−^ mice do not [22,23]. TYRO3^−/−^ mice have an intermediate phenotype [23]. These data indicate a crucial role for TAM RTKs and particularly for AXL in regulation of erythropoiesis in response to acute anemia.

TAM RTK knockout mice have normal hematologic parameters at resting state but impaired erythropoiesis in response to acute anemic stress, indicating that TAM RTKs may play a more prominent role in disease states. While the exact mechanisms of TAM RTK regulation of erythropoiesis have not yet been elucidated, there is evidence that TAM RTK functions are mediated through interplay of Gas6 and erythropoietin (EPO). Gas6 plasma levels are elevated in EPO-resistant patients undergoing hemodialysis [27]. Gas6 is upregulated in mice in response to acute hemolytic stress and in a human erythroblast cell line in response to stimulation by EPO. Treatment of mice with recombinant Gas6 hastens their recovery from both acute hemolytic anemia and acute blood loss anemia. This effect is abrogated in AXL^−/−^ mice but is maintained in a model of chronic anemia secondary to EPO insufficiency [23]. These data suggest that Gas6 functions to increase erythropoiesis in pathological anemia states by increasing signaling through one or more of the TAM RTKs.

### 2.3. Role in Megakaryopoiesis

As with studies of the erythropoietic functions of TAM RTK, most of our understanding of their role in megakaryopoiesis comes from analyses of single, double, or triple TAM RTK knockout mice. Megakaryopoiesis occurs in a number of tightly regulated stages beginning with differentiation of megakaryocyte-erythroid progenitor cells into committed megakaryocyte precursors, then maturation into megakaryocytes, and finally, fragmentation into platelets. Mature megakaryocytes express all three TAM RTKs at relatively high levels [28] and expression of MERTK and AXL on platelets has been independently verified (Figure 1) [29,30,31]. TYRO3 expression on mature platelets has been less well studied and there are some conflicting data [29,30], but platelets from TYRO3^−/−^ mice do have deficiencies in aggregation [28,31,32]. Single or double TAM RTK knockout mice have normal platelet numbers [28,30] but mice deficient in all three receptors have marked thrombocytopenia with platelet levels <50% of controls [28]. This thrombocytopenia can be partially accounted for by the decreased bone marrow megakaryocyte number in triple knockout mice. Interestingly, although double knockout AXL^−/−^/MERTK^−/−^ mice have normal platelet number, they too have decreased bone marrow megakaryocytes. In these mice there are also defects in proplatelet formation potentially due to defects in megakaryocyte maturation as indicated by an underdeveloped demarcation membrane system [28]. However, in triple or AXL^−/−^/MERTK^−/−^ mice splenic megakaryocytopoiesis is increased which may account for the partially preserved platelet numbers. Additionally, there were no obvious deficits in maturation of splenic megakaryocytes, indicating that loss of TAM RTKs in the bone marrow microenvironment may be the mechanism of deficient megakaryocytopoiesis rather than loss of TAM RTKs on megakaryocytes themselves [28]. These data, along with data showing expression of TAM RTKs in bone marrow mesenchymal stromal cells [16,25,26], indicate that TAM RTKs may play a broader role in supporting hematopoiesis.

## 3. TAM Receptors in Hematopoietic Malignancies

### 3.1. Acute Leukemia

Despite advances in patient care and therapeutic options survival rates for patients with acute myeloid leukemia (AML) and acute lymphoblastic leukemia (ALL) are approximately 65% and 85%, respectively, and <50% for adults with acute leukemia [33,34,35], where low survival can be partially attributed to inability to tolerate high doses of cytotoxic chemotherapy and age-related changes in disease biology [36,37,38]. Although children tolerate more aggressive therapy it often comes with frequent hospitalizations [39] where 25% of patients risk suffering severe long-term side effects such as neurocognitive abnormalities, growth hormone deficiencies, infertility, cardiac dysfunction, and secondary malignancies. Additionally, approximately 2/3 of patients experience some late effects of treatment [39,40,41,42].

In AML, FMS-like tyrosine kinase-3 (FLT-3) internal tandem duplication (ITD) mutations are present in approximately 23% and 14% of adult and pediatric AMLs, respectively [43]. The incidence of these mutations and their association with poor prognosis [44,45] prompted the development of FLT3 small molecule inhibitors, including quizartinib and midostaurin. Recent phase III placebo-controlled studies investigating midostaurin in combination with chemotherapy demonstrated improved overall and event free survival in patients treated with combination therapy [46]. While these agents have been effective in the clinic, they provide only short-term remissions as leukemias quickly develop resistance. One study investigating the clinical use of quizartinib reported an average duration of response <12 weeks [47]. In addition, only a subset of AMLs have a FLT3 mutation and targeted treatment options for leukemias without FLT3 mutation remain limited.

#### 3.1.1. MERTK in Acute Myeloid and Acute Lymphoblastic Leukemia

Human MERTK was cloned by functional screening of a human B-lymphoblastoid expression library and later from a human glioblastoma library [17,48]. As described, mature myeloid cells lack expression of MERTK, however 80%–100% of diagnostic pediatric and adult AML patient samples and 85% of AML cell lines aberrantly express MERTK. There is no correlation between surface expression of MERTK and clinical features such as the French-American-British (FAB) or World Health Organization (WHO) classifications, cytogenetic abnormalities, and patient age at time of diagnosis [18]. Similarly, MERTK is ectopically expressed in approximately 50% of T-cell ALL (T-ALL) and 30% of B-cell ALL (B-ALL) patient samples collected at diagnosis [24,49]. In addition, in a transgenic mouse model forced MERTK expression leads to the development of a predominantly T-cell leukemia/lymphoma where transgenic mice have significantly lower tumor-free survival as compared to wild-type C57Bl/6 mice [50].

**Figure 2:**
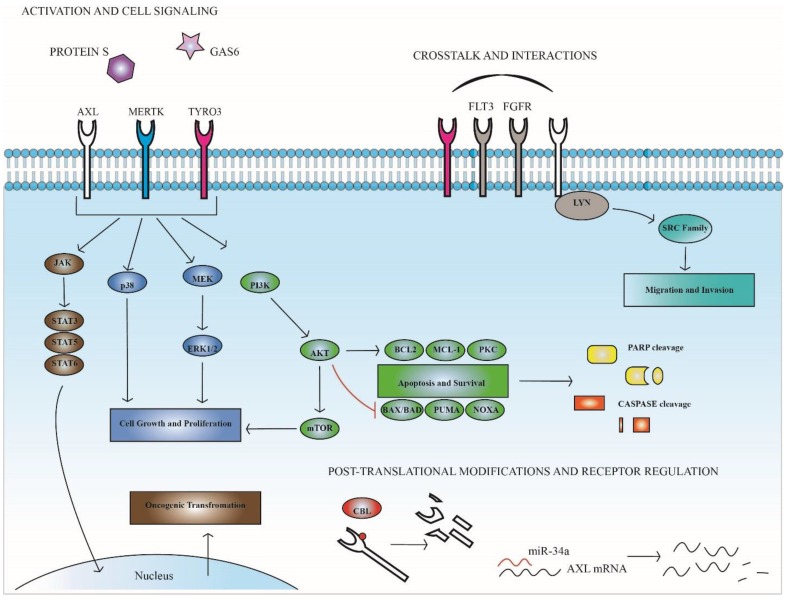
TAM signaling, regulation, and protein interactions in leukemia. TAM receptors signal through pro-survival and anti-apoptotic pathways and also have roles in migration and invasion. Key downstream signaling proteins and their oncogenic functions are depicted above. Specific proteins and response patterns are leukemia subtype dependent. Regulation of AXL by the E3-ligase CBL and miR-34a are also depicted. AXL physically interacts with the proteins FLT3, FGFR, TYRO3 and LYN. The consequences of these interactions are unknown.

MERTK activation leads to phosphorylation of downstream signaling proteins ERK1/2, AKT, p38 and the STAT family of kinases in both AML and ALL cells (Figure 2) [18,49]. ERK1/2 and AKT are known to modulate anti-apoptotic signaling in cancer [51,52], and shRNA-mediated knockdown of MERTK in ALL cell lines decreased expression of genes encoding pro-survival proteins *BCL2L1* (BCL-XL), *PIK3R5* (phosphotidylinositol 3 kinase—PI3K), and *PRKCB* (protein kinase C—PKC). Conversely, shRNA knockdown of MERTK increased expression of genes encoding pro-apoptotic proteins *BAX*, *PMAIP1* (NOXA), and *BBC3* (PUMA) [24]. These changes in downstream apoptotic signaling promote tumor cell survival and inhibition of MERTK using shRNA or small molecule inhibitors induced apoptosis and inhibited colony formation in AML and ALL cell lines and AML patient samples [24,53,54]. In orthotopic cell line and patient-derived xenograft models, MERTK inhibition decreased tumor burden and prolonged survival, implicating MERTK as a therapeutic target [24,49,54]. Additionally, inhibition of MERTK enhanced sensitivity to standard cytotoxic chemotherapies in B-ALL and T-ALL cell lines [24,49], suggesting that clinical application of MERTK inhibitors could be most therapeutically effective in combination with other agents, rather than as a monotherapy.

#### 3.1.2. AXL in Acute Myeloid Leukemia

AXL has also been implicated in AML biology. AXL overexpression in AML was first demonstrated through a retrospective RT-PCR screen of AML patient samples. Researchers observed AXL transcript in 34% of the patient samples [55]. Additionally, expression of AXL has been linked to shorter overall survival in patients with AML [9], regardless of disease subtype or other patient characteristics including patient age [9,55]. The TAM RTK ligand Gas6, which has higher affinity for AXL relative to the other TAM RTKs [56], has been identified as a poor prognostic factor in AML [10], Gas6 is expressed at low levels in AML cells but is also produced in the bone marrow stroma [9]. These observations suggest a role for paracrine signaling between leukemia cells and the bone marrow microenvironment such that together, Gas6 and AXL contribute to tumor cell survival. As might be expected, in the presence of increased Gas6 there was greater AXL activation in AML cell lines. This activation was further increased following treatment with chemotherapy, suggesting the possibility that AXL mediates resistance to chemotherapy in this context. Indeed, treatment of AML cell lines with cytarabine and the AXL inhibitor BGB324 or a ligand sink consisting of the soluble extracellular domains of AXL (sAXL) increased the percentage of apoptotic and dead cells compared to either treatment alone. Additionally, combined treatment with subtherapeutic doses of doxorubicin and BGB324 reduced tumor growth in an AML xenograft model, whereas either single treatment had no effect. Importantly, AXL inhibition is effective regardless of FLT3 mutational status, thereby expanding the patient population that may benefit from a targeted AXL therapy [9,57].

The mechanisms by which AXL inhibition exerts anti-tumor effects are similar to those described for MERTK inhibition in AML and ALL. Roles for downstream signaling through the AKT/PI3K and MAPK pathways have been confirmed (Figure 2) [9,58] and AXL inhibition leads to increased expression of the anti-apoptotic protein PUMA and decreased expression of Bcl-2 [9].

### 3.2. Chronic Lymphocytic Leukemia

#### 3.2.1. AXL and TYRO3 in Chronic Lymphocytic Leukemia

Each year the American Cancer Society compiles a list of cancer incidence, survival, and mortality in the United States. The 2016 report lists chronic lymphocytic leukemia as the second most common form of leukemia, next to AML, and estimates that in this year alone there will be 18,960 new diagnoses [1]. Cytotoxic therapies are used to achieve remissions but typically must be continued long-term and maintaining therapeutic doses in older adults has proven to be difficult in patients with CLL [59]. The recent FDA approval of ibrutinib, a reversible BTK inhibitor, for first-line treatment of patients with CLL provides a novel targeted option for these patients. However, resistance to cytotoxic and targeted therapies is common, highlighting the need for novel treatment options. AXL has been implicated in CLL and is constitutively activated in both patient samples and a CLL-derived cell line [60,61]. Transient siRNA-mediated knockdown of AXL in CLL patient samples led to induction of apoptosis that was replicated by targeted AXL inhibition using small molecule inhibitors, TP-0903 or R428. Treatment of CLL samples with either TKI led to decreased expression of critical anti-apoptotic proteins Bcl-2, Mcl-1, XIAP and increased PARP and caspase 3 cleavage (Figure 2) [61]. Furthermore, even samples with as little as ~20% of cells expressing surface AXL were sensitive to TP-0903 treatment, including samples harboring genetic defects and high-risk mutations [61]. Importantly, AXL inhibition was able to induce apoptosis in CLL cells even in the presence of bone marrow stromal cells [61].

While MERTK expression has not been demonstrated in CLL, immunoblot analysis revealed a substantial increase in expression and activation of TYRO3 in B cells from patients with CLL compared to normal B cells [61]. Co-immunoprecipitation experiments also demonstrated a physical association between AXL and TYRO3 in CLL patient samples but the functional consequences of this interaction are unknown (Figure 2).

AXL is also physically associated with LYN, a Src family kinase (SFK) (Figure 2) [60]. Over expression of LYN in CLL contributes to high levels of protein tyrosine phosphorylation and low response rates to immunoglobulin M ligation therapy [62]. LYN and AXL co-immunoprecipitate and siRNA-mediated AXL knockdown or treatment with the small molecule inhibitor BGB324 (R428), decreased activation of LYN. In contrast, when CLL patient samples were treated with a Src-targeted inhibitor, there was no effect on activation of AXL, despite dramatically reduced phosphorylation of SFKs. These results demonstrate activation of LYN downstream of AXL and implicate LYN as a direct target of AXL kinase activity.

Finally, AXL and fibroblast growth factor receptor 3 (FGFR3) are concurrently expressed and activated in CLL cells (Figure 2) [63]. The FGFR family is responsible for mediating cell processes that are important for oncogenesis, such as migration, proliferation, differentiation, and survival [64]. CLL B cells primarily express FGFR3 [63] and spontaneously secrete basicFGF (bFGF), the ligand for FGFR [65,66]. Constitutive activation of FGFR3 has been observed in CLL B cells coincident with *p*-AXL expression [63]. Studies in non-small cell lung cancer identified cross-talk between AXL and epidermal growth factor receptor [67], suggesting the possibility of a similar interaction between FGFR3 and AXL in CLL. Interestingly, inhibition of AXL with TP-0903 resulted in decreased levels of *p*-FGFR [63]. This effect was phenocopied following siRNA-mediated knockdown of AXL in a breast cancer cell line. Further, stimulation with Gas6 led to an increase in activated FGFR3 without a dramatic effect on AXL phosphorylation, and inhibition with the FGFR-inhibitor TKI-258 did not result in decreased *p*-AXL, implicating AXL activation upstream of FGFR. Consistent with this possibility, AXL and FGFR3 were detected in complex and found to co-localize in CLL cells.

Together these data demonstrate promiscuous interactions between AXL and other kinases that have been implicated in tumorigenesis; however, further work needs to be done in order to fully elucidate the nature and consequences of these interactions, and how they might be effectively targeted in the treatment of CLL.

#### 3.2.2. Regulation of AXL Expression in Chronic Lymphocytic Leukemia

Regulation of AXL expression in CLL is an ongoing area of interest and recently researchers identified a binding site for miR-34 in the untranslated region of AXL. In addition, expression of AXL is substantially increased in samples from patients with a 17p13 mutation in miR-34 compared to samples with no genetic abnormalities, implicating miR-34 as an upstream regulator of AXL expression [68]. miR-34 is directly regulated by p53 and mediates p53-induced apoptosis [69]. Furthermore, miR-34 mutation has been associated with the TP53 mutation [70,71,72] and predicts adverse outcomes in CLL patients [71,72]. Together, these data implicate AXL inhibition by miR-34 as a downstream mediator of p53-induced apoptosis and suggest an additional mechanism for AXL inhibitors in CLL (Figure 2). Consistent with this idea, AXL expression was decreased in patients who had previous doxorubicin treatment relative to patients without treatment [68].

### 3.3. Multiple Myeloma

#### MERTK in Multiple Myeloma

Multiple myeloma (MM) is a hematologic malignancy characterized by monoclonal expansion of malignant plasma cells, anemia, immunodeficiency, and decreased renal function [73]. To date treatment of MM has focused on stem cell transplantation, targeted agents such as the proteosome inhibitor bortezomib, immune modulation with lenalidomide, and standard and high dose cytotoxic chemotherapies. While many patients benefit from these therapies, relapse is common and new therapies are needed [74,75]. Recent areas of interest include immunotherapies that trigger the host anti-tumor immune response and thus far, the results are promising [75,76]. Therapeutic approaches include checkpoint inhibitors, MM vaccines, adoptive T Cell Transfer [77], and monoclonal antibodies. However, these new therapies are at early stages of development and identification of additional novel targets is warranted.

The bone marrow microenvironment contributes significantly to progression of MM [73,78] and given the implications of MERTK inhibition in other leukemias such as AML and ALL, researchers sought to determine its role in MM. Indeed, in bone marrow mononuclear cells from 17 MM patients MERTK and Gas6 mRNAs were increased compared to healthy controls, while AXL and TYRO3 mRNAs were undetectable [79]. Overexpression of MERTK and Gas6 was validated with a much larger cohort of samples using gene expression profiling, with greater than 90% of MM patients expressing MERTK and Gas6. Additionally, five different MM cell lines expressed high levels of MERTK, three of five expressed moderate levels of TYRO3, and AXL expression was low to undetectable. An analysis of MERTK and Gas6 expression in different cytogenetic subgroups of MM yielded no distinct association with low risk versus high-risk MM patient populations.

Similar to MERTK inhibition in AML/ALL, shRNA-mediated MERTK inhibition in MM cell lines decreased proliferation and induced apoptosis following serum starvation, decreased downstream signaling through AKT and ERK1/2, decreased expression of the anti-apoptotic protein Bcl-2, increased cleaved caspase 3, and prolonged survival in a mouse model of systemic myeloma (Figure 2) [79]. Additionally, forced over expression of Gas6 in vivo shortened survival time, and conversely, pharmacologic inhibition of Gas6 by blocking y-carboxylation, which is necessary for Gas6 activity, led to decreased tumor burden and prolonged survival [79]. All of these results validate Gas6 and/or MERTK as potential therapeutic targets in MM. In addition, it has been hypothesized that the other TAM receptors may impact the way the microenvironment communicates with MM cells, but this remains unknown.

## 4. TAM Expression and Function in Therapeutic Resistance

Overexpression of AXL and its role in resistance to targeted and cytotoxic therapies have been shown in several different tumor types, including imatinib-resistant CML and gastrointestinal stromal tumors [80], AML with acquired resistance to FLT3 TKIs [81], cisplatin-resistant ovarian cancer [82], HER-2 positive breast cancer resistant to lapatinib [83], and rhabdomyosarcoma resistant to IGF1R inhibition [84]. While AXL has been implicated in resistance to therapy in a variety of leukemia subtypes and therapeutic settings, the mechanisms of resistance vary. Here we review the role of AXL as a key protein mediating resistance to targeted BCR-ABL inhibition in CML and in resistance to chemotherapy and FLT3 inhibition in AML.

### 4.1. AXL and Resistance in Chronic Myeloid Leukemia

The first and perhaps greatest success story proving the utility of targeted agents for treatment of leukemia was the development of BCR-ABL tyrosine kinase inhibitors for treatment of CML. The pathophysiology of CML is driven by the BCR-ABL fusion protein, which drives over-proliferation of myeloid precursors [85]. Historically, CML was treated with cytotoxic chemotherapy with minimal success, but the introduction of the BCR-ABL TKI Imatinib dramatically changed patient care [86,87,88]. Development of next generation inhibitors is a current priority to treat patients with acquired resistance to front-line BCR-ABL TKIs and to date there are five approved for front line therapy [89]. However, acquired resistance remains the largest hurdle for targeted therapies and new molecules that target BCR-ABL receptors with resistance-conferring mutations or compensatory signaling pathways are needed.

In CML, overexpression and increased activation of AXL has been observed in imatinib-resistant cell line derivatives compared to imatinib-sensitive parental lines [90]. Upregulation of AXL has also been observed in cell lines and patient samples resistant to other small molecule BCR-ABL inhibitors such as nilotinib and PD-166326 [90,91,92]. AXL knockdown via siRNA restored sensitivity to imatinib-resistant CML cell lines in functional assays [90]. Conversely, transfection of wild type AXL into imatinib-sensitive CML cells conferred protection against the anti-leukemic effects of imatinib treatment, such as alteration of cell metabolism and induction of apoptosis, when compared to transfection of kinase dead AXL. These results indicate that imatinib resistance in CML cell lines is mediated, at least partially, by AXL kinase activity.

AXL upregulation in response to BCR-ABL inhibition may be mediated through CBL. CBL is an E3-ligase that stabilizes AXL protein and mRNA, allowing for increased protein expression (Figure 2) [92]. Following CBL depletion in nilotinib-sensitive cells, *AXL* mRNA and AXL protein increased, conversely CBL depletion in sensitive cells resulted in a further increase of AXL protein and mRNA. In cells resistant to nilotinib depletion of CBL returned levels of ubiquinated AXL protein back to those seen in nilotinib sensitive cells [92], indicating AXL regulation by CBL-mediated ubiquination and subsequent degradation of AXL protein. Consistent with these results, in primary hematopoietic cells forced expression of AXL conferred resistance against nilotinib treatment. Further, CBL decrease in CD34^+^ cells results in increased AXL and is associated with nilotinib resistance. Other studies have implicated PKCα and β as regulators of AXL expression based upon the observation that treatment of imatinib-resistant CML cell lines with a PKC inhibitor leads to decreased AXL [90]. Similarly, dual siRNA mediated silencing of PKCα/β decreased AXL and overexpression of PKCα/β increased AXL in imatinib-sensitive cells. These results suggest a mechanism by which PKCα/β are responsible for increased transcription of AXL and the E3-ligase CBL then stabilizes AXL mRNA and protein, leading to increased AXL expression, increased downstream survival signaling, and ultimately therapy resistance.

### 4.2. AXL and Resistance in Acute Myeloid Leukemia

As discussed previously, studies have shown AXL overexpression and constitutive activation in AML cell lines and primary AML patient samples, and AXL has also been implicated in AML with acquired resistance to chemotherapy and/or targeted FLT3 inhibition. For instance, in an analysis of four paired diagnostic and relapsed samples collected from patients who developed resistance to a drug regime containing doxorubicin and cytarabine, all four of the resistant samples expressed higher levels of AXL [58]. Similarly, in an AML cell line chemotherapy treatment led to a dose-dependent induction of AXL expression, and stimulation with Gas6 conferred resistance to doxorubicin, VP16, and cisplatin treatment which was reversed by addition of the ligand trap, sAXL [58].

In studies investigating AXL in FLT3-ITD AML, targeted AXL inhibition diminished cell growth in cell lines and xenograft models and AXL expression was necessary for constitutive activation of FLT3 (Figure 2) [57], suggesting a role for AXL in FLT3-ITD AML. In addition, treatment of a FLT3-ITD AML cell line and patient sample with the FLT3 inhibitors PKC412 or quizartinib resulted in increased activation of AXL and treatment with PKC412 increased signaling through pathways downstream of AXL, including ERK1/2, AKT, and STAT5 [81]. AXL activation was inhibited by MEK/ERK and PI3K targeted inhibitors, indicating that the activation of AXL is mediated mainly through these pathways. Evaluation of PKC412 treated cells undergoing apoptosis showed increased AXL activation in the live population compared to the Annexin^+^ cells. Interestingly, when PKC412-resistant AML cell lines were treated with the AXL inhibitor TP-0903 they were resensitized to FLT3 targeted therapy both in vitro and in vivo. The in vitro results were replicated using sAXL and shRNA-mediated AXL knockdown [81].

## 5. Therapeutic Targeting of TAM Receptors

### 5.1. Tyrosine Kinase Inhibitors

Given the validation of TAM RTKs as efficacious targets in many different tumor types, recent research has focused on developing targeted inhibitors for use in the clinic. While there are a number of small molecule inhibitors in preclinical and clinical development that have activity against TAM RTKs [6,93,94], many of these were not specifically designed as such and preferentially target other kinases. Here we review compounds rationally designed to target TAM RTKs (Table 1).

#### 5.1.1. AXL

##### BGB324 (R428)

BGB324, also known as R428, is the first selective small molecule inhibitor of AXL to enter clinical development [95]. The compound precursor was first identified in a high-throughput screen and modifications were introduced based on structure-activity relationships (SAR) to enhance selectivity [96]. BGB324 is 50-fold selective for AXL relative to MERTK in cell-based assays and >100-fold selective over TYRO3. In mice, a single dose of 75 mg/kg resulted in a C_max_ of 6.8 µM with a plasma half-life of 13 h. Additionally, in two models of murine breast cancer, treatment with BGB324 blocked known AXL-mediated metastatic functions and synergized with cisplatin, a cytotoxic chemotherapy, to prevent liver micrometastases [96].

BGB324 has been extensively evaluated in preclinical models of both CLL and AML [9,60], and a phase I multicenter trial of BGB324 in combination with cytarabine is currently recruiting patients with AML [97]. As described above, AXL is constitutively active in CLL, and in vitro culture of primary CLL B cells with BGB324 inhibited AXL phosphorylation and induced apoptosis. Similarly, in AML cell lines and primary patient samples, culture with BGB324 inhibited AXL activation, induced apoptosis, and enhanced chemosensitivity to doxorubicin and cytarabine, regardless of FLT3 mutational status [9]. Treatment with BGB324 resulted in tumor regression in mice with subcutaneous xenografts of the human FLT3-ITD AML cell line MV4-11 [9]. In contrast, apoptosis was not induced when non-leukemic B cells, T cells, or NK cells were cultured with BGB324 [60] and AXL-negative patient samples and healthy bone marrow mononuclear cells were similarly resistant to BGB324 [9], underlining the potential for selective targeting of AXL as a therapeutic strategy in leukemia.

The promising results of preclinical studies such as these supported clinical development of BGB324 and in 2013, a phase I study in healthy volunteers was initiated to demonstrate safety and tolerability and provide pharmacokinetic data [95,98]. In 2015, a phase I multicenter trial of BGB324 as monotherapy and in combination with cytarabine in patients with relapsed or refractory AML or as monotherapy in patients with high-risk myelodysplastic syndrome (Multicenter Open-label Study of BGB324 as a Single Agent and in Combination With Cytarabine in Patients With AML, NCT02488408) [97].

##### TP-0903

TP-0903 is another compound in preclinical and clinical development as a potent AXL inhibitor with an IC_50_ of 27 nM in enzymatic assays. In biochemical assays, TP-0903 potently inhibits phosphorylation of AXL and its downstream target AKT with EC_50_ (effective concentration) values of 222 and 350 nM, respectively [99]. However, it is also potent against TYRO3 and MERTK and the kinases Aurora A and B. Additionally, TP-0903 has a much lower IC_50_ in cell viability assays indicating that functional effects may be mediated by off-target inhibition and not solely through AXL [93,99]. Despite this, evaluation of TP-0903 as an AXL inhibitor in solid tumors, AML, and CLL has moved forward [93].

In preclinical studies, treatment of CLL and AML cell cultures with TP-0903 resulted in decreased AXL phosphorylation and induction of apoptosis at concentrations achievable in vivo [61,81]. Interestingly, circulating CLL cells from patients treated for up to 4 weeks with the reversible BTK inhibitor ibrutinib retained ex vivo sensitivity to TP-0903 [100], but combined treatment with TP-0903 and ibrutinib was antagonistic in 7 of 11 patient samples [61]. The potential mechanism of this unfavorable interaction is unclear but this effect was not observed when TP-0903 was combined with another BTK inhibitor, TP-4216. Further investigation is needed prior to moving combination therapy with BTK and AXL inhibitors into clinical trials in CLL. As previously noted, in a FLT3-ITD AML cell line resistant to FLT3 inhibition, treatment with TP-0903 resensitized the cells to two different FLT3 small molecule inhibitors [81], implicating dual inhibition of FLT3 and AXL as an intriguing therapeutic strategy in AML.

A first in human phase I trial of TP-0903 is planned to open in late 2016 in patients with advanced solid tumors with future clinical development aimed towards patients with ibrutinib-resistant CLL and FLT3 inhibitor-resistant AML [97,101].

##### ASP2215 (gilteritinib)

ASP2215 is a dual FLT3/AXL inhibitor that is currently in phase III clinical trials in AML [97,102]. ASP2215 has an IC_50_ of 0.7 nM against AXL [93] and 0.29 nM against FLT3 [102] in enzymatic assays, potently inhibits phosphorylation of FLT3 in a FLT3-ITD^+^ AML cell line, and prolongs survival in orthotopic xenograft models of FLT3-ITD^+^ AML. Importantly, in a colony-forming assay treatment with ASP2215 was 100-fold more potent in the AML cell line compared to normal human granulocyte-macrophage precursors, suggesting a wide therapeutic window [102]. A first-in-human phase I/II trial was conducted in patients with relapsed or refractory AML and ASP2215 was well-tolerated, with favorable PK and once daily oral dosing [103,104,105]. Pharmacodynamic evaluation has thus far focused on inhibition of FLT3 phosphorylation and assessment of AXL inhibition has not been described. Additionally, in this trial the overall response rate for patients with FLT3 activating mutations was 57%, compared to only 11% in FLT3 wild-type patients [104]. ASP2215 trials are ongoing, both in newly diagnosed AML patients and relapsed/refractory populations; however, without regard for AXL expression, likely due to the current lack of biomarkers to predict response to AXL inhibition in leukemia [97].

#### 5.1.2. MERTK

Although no MERTK-targeted small molecule inhibitor has been advanced into clinical trials, there are a number in preclinical development for leukemia. One of the first reported MERTK selective inhibitors was UNC569, which potently inhibited MERTK phosphorylation in B- and T-ALL cell lines [106,107] and had therapeutic efficacy in a zebrafish model of T-ALL [107]. Unfortunately, UNC569 has off-target activity towards hERG and suboptimal potency against MERTK [108]. Subsequent derivatives, UNC1062 and UNC1666, have decreased activity towards hERG and increased potency against MERTK but neither is suitable for translational studies due to pharmacokinetic profiles [53,108].

To optimize MERTK inhibitors for clinical use, further modifications of UNC1062 yielded UNC2025 [109]. UNC2025 is highly potent against MERTK with an IC_50_ of 2.7 nM and ≥45-fold selective over TYRO3 or AXL in cell-based assays. A single oral dose of 3 mg/kg yielded a C_max_ of 1.6 µM with T_1/2_ of 3.8 h in mice. The compound has been well-tolerated with minimal and manageable toxicities in animal models [110]. Additionally, it mediates near complete inhibition of MERTK phosphorylation in the bone marrow of leukemia-bearing mice [109,110]. Treatment with UNC2025 inhibited MERTK-expressing leukemia cell proliferation and colony-forming potential, induced apoptosis, and in some cases induced polyploidy [110]. In cell line xenograft models of MERTK-dependent B-ALL, treatment with UNC2025 increased survival both alone and in combination with cytotoxic chemotherapy. Moreover, in a patient-derived AML xenograft model, treatment with monotherapy was sufficient to induce disease regression in the bone marrow, blood, and spleen. In addition, UNC2025 mediated potent anti-leukemia activity in approximately one third of 261 primary leukemia patient samples and the AML and T-ALL subsets were particularly sensitive, with 40% to 50% of samples responding to treatment with UNC2025, suggesting that a large portion of patients with leukemia could benefit from treatment with UNC2025 or similar compounds and supporting continued clinical development. Toward this end, MRX-2843, an analog to UNC2025 with a minor side chain substitution and similar PK properties and toxicity profile [54,109], was recently approved by the Food and Drug Administration (FDA) for clinical testing. Like UNC2025, MRX-2843 induced apoptosis, and inhibited colony formation in MERTK-expressing leukemia cell cultures. Additionally, MRX-2843 prolonged survival 2–3 fold compared to vehicle controls in both cell line and patient derived xenograft (PDX) models of AML. Importantly, these compounds are 10–20 fold more potent against leukemia cells compared to normal human cord blood mononuclear cells in colony formation assays, indicating potential for a wide therapeutic window [54,110].

Interestingly, UNC1666, UNC2025, and MRX-2843 are dual-specificity kinase inhibitors and are also highly potent against FLT3. UNC1666 and MRX-2843 both inhibited colony formation in FLT3-ITD patient samples [53,54], and MRX-2843 prolonged survival in orthotopic PDX models of FLT3-ITD AML. MRX-2843 potently inhibited activation of FLT3 in cell-based assays, even in the presence of clinically-relevant D835Y and F691L FLT3 point mutations, which confer resistance to many of the currently available FLT3 TKIs [54,111]. As previously described, upregulation of AXL has been implicated as a mechanism of resistance to FLT3 inhibition in AML and a dual FLT3/AXL inhibitor (ASP2215) is currently in clinical development, making further evaluation of MRX-2843 as a dual MERTK/FLT3 inhibitor particularly attractive. Finally, MRX-2843 has recently received FDA Investigational New Drug (IND) status and will be the first-in-class MERTK inhibitor used in clinical trials.

#### 5.1.3. TYRO3

Unlike AXL or MERTK, there is limited published data on small molecule inhibitors selective for TYRO3. A number of SAR studies describing optimization of selective inhibitors have been published [112,113,114] but there have been no further reports on preclinical development of the lead compounds.

### 5.2. Biologic TAM RTK Inhibitors

Biologic TAM RTK inhibitors, such as monoclonal antibodies, ligand sinks, and aptamers [115], are also in preclinical development and potentially have the advantage of improved selectivity relative to small molecule inhibitors. To date, the majority of these studies have focused on solid tumor models, but the known roles for TAM RTKs suggest they will be applicable in leukemia as well. Monoclonal antibodies directed at the extracellular domains of TYRO3 [116], MERTK [117,118], or AXL [119,120,121,122] have all been developed and, in general, function by increasing receptor internalization and degradation. A monoclonal antibody that recognizes the extracellular domain of MERTK, Mer590, impeded activation of MERTK, inhibited colony formation, and synergistically enhanced carboplatin-induced apoptosis in NSCLC cells [117]. Additionally, Mer590 inhibited migration in glioblastoma multiforme cells [118]. A number of anti-AXL antibodies have also been described, including Mab173, 12A11, D9, and E8. Mab173 inhibited invasion and induced tumor regression in mouse models of Kaposi sarcoma [119], 12A11 inhibited growth of subcutaneous NSCLC xenografts [120], and D9 and E8 inhibited tumor cell migration and viability and prolonged survival in orthotopic murine models of pancreatic cancer [121]. Additionally, the anti-AXL antibody YW327.6S2 recognizes both human and murine AXL and is thus useful for preclinical evaluation of the effects of AXL inhibition in the tumor microenvironment [122]. Indeed, in NSCLC and breast cancer models, YW327.6S2 has direct anti-tumor effects and also enhances the effects of an anti-VEGF antibody on the tumor-associated vasculature. Although there are no published studies describing the use of these monoclonal antibodies in leukemia, they are effective in solid tumor models and are an attractive area for further research in hematologic malignancies.

Biologic agents that target TAM RTK ligands have also been effective in preclinical models. Decoy receptors have been engineered by fusing the extracellular domains of the TAM RTKs with the Fc domain from human IgG [56,123,124]. These receptors function as ligand sinks by binding to TAM RTK ligands and thereby inhibiting ligand-induced activation. To enhance the affinity for Gas6 binding, decoy receptors with engineered mutations in the AXL extracellular domain have been developed and treatment with these high affinity proteins decreased tumor metastasis in murine models of ovarian cancer and breast cancer [124]. Similarly, treatment with a Gas6 neutralizing antibody decreased tumor growth in a pancreatic ductal adenocarcinoma model [125]. Importantly, FLT3-ITD^+^ AML cell cultures, treatment with AXL-Fc inhibited phosphorylation of AXL and decreased cell number by altering cell cycle progression and inducing apoptosis [57,81]. AXL-Fc treatment led to decreased tumor growth and prolonged survival in subcutaneous AML xenograft models [57], demonstrating the potential therapeutic application of TAM RTK ligand traps in leukemia.

## 6. Future Areas of Research

Over the past decade there has been a rapid increase in our appreciation of TAM biology, and particularly, in the number of TAM-selective therapeutic strategies. These advancements have opened the door for many new areas of interest with respect to the roles of TAM RTKs in leukemia. One area of active focus in both leukemia and solid tumors is in harnessing the patient’s immune system to provide an anti-tumor effect. Specifically in leukemia, immunomodulatory strategies such as chimeric antigen receptor (CAR)-T-cell therapies, bi-specific T-cell engager antibodies, and immune checkpoint blockade have had impressive results [126] and targeted inhibition of TAM RTKs in the immune system and tumor microenvironment represents an attractive alternative strategy for immune regulation. The TAM RTKs play important roles in negative regulation of the immune system [14] and there is evidence in murine tumor models that they function to promote a pro-tumorigenic phenotype [127,128,129]. Tumor growth is delayed and metastasis is decreased in MERTK^−/−^ syngeneic murine models of breast cancer, melanoma, and colon cancer compared to wild-type controls [127]. Additionally, in a transgenic model of mammary gland carcinoma, reconstitution of lethally irradiated mice with MERTK^−/−^ marrow again reduced tumor growth, highlighting the importance of MERTK in the hematopoietic system for tumor development [127]. These data demonstrate the potential for a dual therapeutic strategy with both direct anti-tumor effects and enhanced anti-tumor immunity mediated by inhibition of a single TAM RTK target. Given the recent dramatic successes of immunomodulatory therapies in hematologic and other malignancies, this should be an important area of further exploration.

With the advent of TAM RTK inhibitors into clinical trials, it will be important to understand mechanisms of resistance to TAM inhibition. A particular area of focus is the role of the bone marrow microenvironment in leukemia. Bone marrow stromal cells have long been implicated in protection of leukemia cells from therapeutic effects mediated by both cytotoxic chemotherapies and TKIs [130,131,132]. In terms of resistance to TAM RTK inhibition, this protection may be mediated through upregulation of Gas6 ligand, as multiple lines of evidence implicate Gas6 in therapeutic resistance in the bone marrow niche. Gas6 produced by stromal cells promotes the colony forming potential of hematopoietic stem cells [133] and has more recently been identified as an independent poor prognostic factor in AML [10], implying a role for Gas6 in maintaining the leukemia stem cell population. Additionally, in pre-B ALL, the Gas6/MERTK axis regulates homing to the bone marrow niche [134]. Indeed, primary human bone marrow stromal cells protected CLL cells from induction of apoptosis with the AXL inhibitor BGB324 [60]. Therapeutic strategies to overcome this resistance include combined treatment with ligand traps to sequester Gas6 or with bone marrow mobilizing agents such as the CXCR4 antagonist, plerixafor, which has been shown to enhance sensitivity to both cytotoxic chemotherapy and targeted agents in ALL models [132,135].

Finally, as described above, the roles of both AXL and MERTK in leukemia have been well described, but less is known about TYRO3. However, TYRO3 is aberrantly expressed in AML [10,136] and multiple myeloma [137] patient samples, and shRNA-mediated knockdown of TYRO3 in a melanoma model has a negative impact on cell survival in the majority of cell lines tested [116]. Thus, TYRO3 most likely contributes to leukemogenesis, resistance to therapy, or both, but the extent of its role is unknown. Further investigation into the role of TYRO3 in hematologic malignancies is warranted.

## 7. Concluding Remarks

In summary, targeted inhibition of members of the TAM (TYRO-3, AXL, MERTK) family of receptor tyrosine kinases has recently been investigated as a novel strategy for treatment of hematologic malignancies. The role of TAM RTKs in innate immune control mediated by regulation of efferocytosis and the inflammatory cytokine response has previously been well described. However, TAM RTKs also function to regulate erythropoiesis and they play important roles in platelet formation and activation. Further, the TAM family receptors are aberrantly or ectopically expressed in many hematologic malignancies, including acute myeloid leukemia, B- and T-cell acute lymphoblastic leukemia, chronic lymphoblastic leukemia, and multiple myeloma. TAM receptors contribute to leukemic phenotypes through activation of oncogenic signaling pathways leading to increased cell survival and proliferation, and resistance to both cytotoxic chemotherapeutics and targeted agents. The role of TAM receptors in chemoresistance makes them particularly attractive therapeutic targets and a number of translational strategies for TAM inhibition have been developed, including tyrosine kinase inhibitors (TKIs), ligand traps, and monoclonal antibodies. AXL-selective TKIs have shown promise in preclinical models, particularly in combination with targeted BCR-ABL and FLT3 inhibition in CML and AML, respectively, and clinical trials are currently underway combining AXL inhibition with standard chemotherapy in AML. Although efforts to target TAM receptors have thus far been primarily focused on AXL, MERTK-selective small molecule inhibitors have recently been described, are effective in preclinical models of ALL and AML, and are progressing toward clinical development. Emerging areas of research include modulation of TAM receptors to enhance anti-tumor immunity, mechanisms of resistance to TAM inhibition, and potential roles for TYRO-3 in leukemogenesis.

## Figures and Tables

**Table 1:** TAM receptor small molecule inhibitors in development.

Compound	IC_50_ Values						Other Targeted Kinases
	TYRO3		AXL		MERTK		
	Enzymatic	Cell-Based	Enzymatic	Cell-Based	Enzymatic	Cell-Based	
BGB324	200 nM	>1400 nM	14 nM	14 nM	220 nM	700 nM	ABL, RET, TIE2, FLT3
TP-0903	<200 nM		27 nM	222 nM	<200 nM		AURKA, AURKB, JAK2, ALK, ABL1
ASP2215			0.7 nM		2.9 nM		FLT3, LTK, ALK
UNC2025	17 nM	301 nM	14 nM	122 nM	0.74 nM	2.7 nM	FLT3, TRKA, KIT
MRX-2843	17 nM		15 nM		1.3 nM		FLT3, TRKA
